# Pathogenesis and Virulence of *Candida albicans* and *Candida glabrata*

**DOI:** 10.3390/pathogens9090752

**Published:** 2020-09-16

**Authors:** Mariana Henriques, David Williams

**Affiliations:** 1 Department of Biological Engineering, University of Minho, 4715-338 Braga, Portugal; 2 Oral and Biomedical Sciences, School of Dentistry, College of Biomedical and Life Sciences, Cardiff University, Heath Park, Cardiff CF14 4XY, UK

**Keywords:** *Candida albicans*, *Candida glabrata*, *Candida* infection, virulence factors, pathogenicity

## Abstract

Fungal infections in humans have historically received comparatively less attention than those caused by bacteria and viruses. This may, in part, stem from the relative differences in infection prevalence. However, the more widespread use of immunosuppressive therapy, invasive surgery and medical devices in modern medicine has resulted in a more frequent occurrence of human fungal infection. There are a number of fungi that can cause human disease. However, it is arguably a species of the genus *Candida* that are most often encountered. There are over 150 *Candida* species that are widely encountered in the environment and in animal hosts, however, only a small number are opportunistic pathogens of humans. *Candida albicans* is a polymorphic yeast-like fungus and undoubtedly the species most often recovered from human infection. As such, the virulence of *C. albicans* and its susceptibility to antifungal agents are often investigated. More recently, the prevalence of infections caused by non-*C. albicans* *Candida* species have increased and, amongst these, infections caused by *Candida glabrata* have received attention given its often-higher tolerance to frequently used antifungals exhibited by this species. The papers presented in this Special Issue have focused on aspects relating to host responses to *Candida* infection, the efficacy of novel therapeutic agents and also treatment regimes. The papers highlight novel findings in their respective areas, whilst also highlighting the need for further research in these key and largely under-researched areas of candidoses.

Several *Candida* species are considered to be opportunistic pathogens in humans. Generally, these fungal microorganisms colonise the mucosal surfaces of the body without causing damage. However, a changing environment can lead to microbial dysbiosis with an accompanying switch in the lifestyle of the *Candida* from a harmless commensal to an organism that causes disease. The manifestation of infection is frequently mild, and this is especially the case when topical infection occurs. However, in individuals who are severely immunocompromised, systemic infections can develop, and whilst these are relatively uncommon, they are associated with high patient mortality. The prognosis of a candidosis is dependent on numerous factors that relate to the virulence of the microorganism, the host’s response and the susceptibility of the *Candida* to therapeutic intervention. Compared with bacteria, the levels of our understanding of the virulence, host immune responses and extent of treatment options are limited. This Special Issue collates six papers that cover aspects relating to all of these research areas.

Aspects of the host immune response to *Candida* have been considered by two articles [1,2] in this Special Issue. The first of these, by Braunsdorf et al., [1] is an elegant review of host immunity against *C. albicans*. The review acknowledges that whilst impaired host immunity has a significant role in the development of candidoses, it is also apparent that strain differences can also affect this process and thus influence whether commensal colonisation by *C. albicans* progresses to an infection state. Such strain-specific differences can lead to altered regulation at the genomic, genetic, and epigenetic levels and this is made more complex by additional environmental factors.

As previously indicated, the most prevalent forms of candidoses are topical infections, and these frequently arise on moist mucosal surfaces, as found in the mouth and vagina. One particularly important form of oral candidosis is chronic hyperplastic candidosis (CHC) given its suggested link with malignant cellular transformation. To better understand host immune responses in this infection, Williams et al. [2] employed immunohistochemical staining to delineate the inflammatory cell infiltrate mounted by the host in response to hyphal invasion of the epithelium. In CHC, the mean proportion of T-cells (CD3^+^) in tissues was 15.6%, with the majority found in the lamina propria. B-cells (CD20^+^) were, comparatively, present in much lower proportions (1.8%). Regarding T-cells, T-helper cells (CD4^+^) were more prevalent than cytotoxic T-cells (CD8^+^). The study concluded that a T-helper cell-dominated immune response occurred in CHC that was likely driven by the invading *Candida* and was notably distinct from an inflammatory cell profile encountered in control squamous cell papilloma tissue sections.

One of the difficulties in the management of candidoses is the limited number of effective antifungal agents that do not have an associated host cell toxicity, or risk the development of resistance. There is, therefore, a need for new and alternative antifungal agents that are also active against recalcitrant biofilms of the fungus. Increasingly, natural products such as those derived from plants, are being assessed for their antifungal effects. Such products frequently comprise a complex mixture of compounds that may each have antimicrobial effects on different microbial targets. As such, synergy may be evident with a reduced likelihood of resistance development. In this Special Edition, two articles were focused on the evaluation of such natural compounds. In the study by Guwca et al., [3] extracts of propolis (EEPs), obtained from Polish apiaries, were evaluated for their antifungal effects against *C. albicans*, with minimum fungicidal concentrations (MFCs) evident between 0.08–1.25% (*v*/*v*). Synergistic antifungal effects against *C. albicans* occurred when EEPs were used in combination treatments with both fluconazole and voriconazole. At sub-inhibitory concentrations, it was evident that the yeast-to-hyphal transformation of *C. albicans* was reduced. EEPs were also effective against *C. albicans* biofilms on polystyrene surfaces at concentrations between 0.04% (*v*/*v*) and >1.25% (*v*/*v*), as well as *C. glabrata* and *C. krusei* biofilms on polyvinyl chloride and silicone catheters. 

Similarly, in the study of Serra et al., [4], the anti-*C. albicans* activity of commercially available essential oils was compared with chlorhexidine and triclosan. Against planktonic *C. albicans*, all tested essential oils were inhibitory with minimum inhibitory concentrations (MICs) ranging between 0.06 (*v*/*v*) and 0.4% (*v*/*v*). Of note, biofilms exhibited an elevated resistance to these agents with minimum biofilm eradication concentrations reported at >1.5% (*v*/*v*). Importantly, for the essential oils, cytotoxicity towards murine fibroblasts was at concentrations lower than the MICs which would be a limitation when developing these new treatments. 

One potential solution to reduce host cell toxicity from antifungal agents is to develop alternative formulations that retain desired antifungal properties whilst reducing the damage to host cells. An excellent example of this is the use of a lipid formulation of amphotericin (AmB) such as AmBisome^®^, Abelcet^®^ and AmB-L. The latter is a liposomal formulation complexed with distearylphosphatidyl glycerol (DSPG) and cholesterol which was investigated, along with the deoxycholate (Deox) amphotericin formulation, for activity against both *C. albicans* and *C. glabrata* in a study by Rodrigues and Henriques [5]. Experiments revealed that *C. glabrata* was generally more tolerant to the lipid and Deox formulations of AmB. When tested against biofilms, the lipid formulation was deemed to have a better therapeutic potential than AmB-Deox, as effective concentrations were at 10% of the maximum daily dose.

Imbert et al. [6], assessed the difficulties in preventing the development of candidemia that is associated with *Candida* biofilms on vascular catheters. One potential approach is to use very high concentrations of antifungal in antifungal lock therapies to sterilise catheters. This study evaluated existing data in the literature to determine the efficacy of a range of antifungal solutions used for lock therapy. It was concluded that whilst caspofungin, micafungin, anidulafungin and L-AmB were good candidates for lock therapy, they had to be used in combination with a systemic therapy. 

## References

[1] Braunsdorf C., LeibundGut-Landmann S. (2018). Modulation of the Fungal-Host Interaction by the Intra-Species Diversity of *C. albicans*. Pathogens.

[2] Williams A., Williams D., Rogers H., Wei X., Lewis M., Wozniak S., Farnell D., Jones A. (2019). Immunohistochemical Expression Patterns of Inflammatory Cells Involved in Chronic Hyperplastic Candidosis. Pathogens.

[3] Gucwa K., Kusznierewicz B., Milewski S., Van Dijck P., Szweda P. (2018). Antifungal Activity and Synergism with Azoles of Polish Propolis. Pathogens.

[4] Serra E., Hidalgo-Bastida L.A., Verran J., Williams D., Malic S. (2018). Antifungal Activity of Commercial Essential Oils and Biocides against *Candida albicans*. Pathogens.

[5] Rodrigues C.F., Henriques M. (2017). Liposomal and Deoxycholate Amphotericin B Formulations: Effectiveness against Biofilm Infections of *Candida* spp.. Pathogens.

[6] Imbert C., Rammaert B. (2018). What Could Be the Role of Antifungal Lock-Solutions? From Bench to Bedside. Pathogens.

